# Noradrenergic Modulation of Cognition in Health and Disease

**DOI:** 10.1155/2017/6031478

**Published:** 2017-05-03

**Authors:** Olga Borodovitsyna, Matthew Flamini, Daniel Chandler

**Affiliations:** Department of Cell Biology and Neuroscience, Rowan University School of Osteopathic Medicine, Stratford, NJ 08084, USA

## Abstract

Norepinephrine released by the locus coeruleus modulates cellular processes and synaptic transmission in the central nervous system through its actions at a number of pre- and postsynaptic receptors. This transmitter system facilitates sensory signal detection and promotes waking and arousal, processes which are necessary for navigating a complex and dynamic sensory environment. In addition to its effects on sensory processing and waking behavior, norepinephrine is now recognized as a contributor to various aspects of cognition, including attention, behavioral flexibility, working memory, and long-term mnemonic processes. Two areas of dense noradrenergic innervation, the prefrontal cortex and the hippocampus, are particularly important with regard to these functions. Due to its role in mediating normal cognitive function, it is reasonable to expect that noradrenergic transmission becomes dysfunctional in a number of neuropsychiatric and neurodegenerative diseases characterized by cognitive deficits. In this review, we summarize the unique role that norepinephrine plays in prefrontal cortical and hippocampal function and how its interaction with its various receptors contribute to cognitive behaviors. We further assess the changes that occur in the noradrenergic system in Alzheimer's disease, Parkinson's disease, attention-deficit/hyperactivity disorder, and schizophrenia and how these changes contribute to cognitive decline in these pathologies.

## 1. Introduction

The monoamine transmitter norepinephrine (NE) is synthesized and released by several small brainstem nuclei, and it has important modulatory roles in a number of forebrain functions. While classically thought to be primarily involved in sensory signal detection [1, 2] and general arousal and alertness in the waking state [3–5], more recent evidence suggests that NE plays important roles in behavior and cognition, such as attention [6–10], behavioral flexibility [11–14], and learning and memory [15–20]. Although disruption of these cognitive functions is not diagnostic of one specific disease state, it is symptomatic in a host of neuropsychiatric and neurodegenerative disorders [21–27]. Importantly, there is strong evidence linking dysfunction of the noradrenergic system to many such conditions, including depression [28, 29], anxiety [30, 31], attention-deficit hyperactivity disorder (ADHD) [32–36], schizophrenia [37–39], autism [40], Parkinson's disease [29, 41, 42], and Alzheimer's disease [27, 43, 44]. NE in the prefrontal cortex (PFC) and hippocampus is particularly important in the maintenance of multiple discrete behavioral and cognitive functions in both health and disease [7, 8, 15, 19, 20, 45–48]. It has been demonstrated that manipulations of the NE system in hippocampal and prefrontal regions are capable of selectively altering discrete aspects of behavior. For example, NE within the medial PFC is required for extradimensional shifting, a higher order measure of behavioral flexibility, but not for other measures of behavioral flexibility that are dependent upon the integrity of other neural substrates [7, 8].

NE is also required for hippocampal memory consolidation and retrieval [16, 17, 48]. Because NE has been identified as a potent modulator of various measures of prefrontal [45, 46, 49–55] and hippocampal function [15, 17, 47, 48], it is highly important to understand how the locus coeruleus (LC) and forebrain noradrenergic signaling adapt in various disease states. For example, early sensory deficits that occur in Alzheimer's and Parkinson's diseases that precede major cognitive decline and motor deficits might be related to deficits in noradrenergic signaling [43, 44, 56] due to its facilitatory role in sensory signal discrimination [1, 51, 57–59]. Indeed, LC neurons are known to degenerate in both of these conditions [27, 29, 42, 56], potentially limiting forebrain noradrenergic facilitation of sensory perception. In this review, we will summarize the role and actions of NE in PFC and hippocampus and how it contributes to behavior and cognition. Furthermore, we will consider how both the LC proper and forebrain noradrenergic transmission are known to change in some disease states characterized by disordered cognition and how behavioral deficits in a multitude of neuropsychiatric and neurodegenerative diseases might allude to dysfunction of the noradrenergic system (Table 1). Recognition of the noradrenergic system as a major contributor to normal cognition and that its dysfunction can precipitate cognitive impairment represents an important step forward in identifying causes of and potential therapies for a number of pathological states.

## 2. Role of Norepinephrine in Prefrontal Cortical and Hippocampal Function and Behavior

Norepinephrine was originally thought to play a principal role in promoting waking due to the correlation between LC discharge rate and an animal's behavioral state [60–62] and the fact that artificial activation of LC promotes a forebrain EEG associated with waking in both the cortex and hippocampus [3–5]. A somewhat more specialized view for the role of NE came to light when it was shown that NE and LC activation can modulate the response properties of sensory neurons following stimulation in a dose-dependent manner [1, 2, 57]. Therefore, in addition to promoting waking, NE at particular levels might facilitate detection of sensory stimuli by priming sensory neurons. For example, LC stimulation and drugs that promote noradrenergic transmission have both been shown to increase responsiveness to visual stimulation in primary sensory neurons in the lateral geniculate nucleus [9, 10, 63, 64]. Through these combined actions, NE may have procognitive effects simply by rendering animals more alert and more sensitive to salient sensory stimuli in their environments. This is important because a major component of cognition is attention: the ability to ignore irrelevant sensory stimuli and focus on those that are behaviorally relevant. It is known that LC is activated by salient sensory stimuli that predict reward and that these responses are plastic such that they shift to new reward predictive stimuli when previously useful stimuli lose their predictive value [14, 49, 65–67].

These observations suggest that LC maintains an active role in regulating sustained and flexible attention that is more complex than simply increasing sensory neuronal responsiveness to nonspecific stimuli. Thus, if all sensory neurons became more sensitive to stimulation through the actions of NE when LC was activated by the reward predictive stimulus, it would be difficult for an animal to attend the relevant stimulus and ignore the irrelevant stimuli. Therefore, there is likely a degree of filtering or selection, either by LC or its terminal fields, that allows LC to respond preferentially to the relevant stimulus. A likely sight for this selection is in PFC neurons, which likewise show preferential responsiveness to task-relevant stimuli [46, 49]. Interestingly, the psychostimulant methylphenidate, a NE/dopamine reuptake inhibitor, simultaneously improves cognitive functions such as attention and working memory as well as prefrontal neuronal responsiveness [68]. It also preferentially increases NE concentration in PFC compared to other LC terminal fields [68–70]. These findings suggest a unique relationship between NE in PFC and cognition. Indeed, lesion studies have shown that denervation of NE fibers, but not cholinergic fibers, in medial PFC impairs extradimensional shifting, which can be rescued by administration of the selective NE reuptake inhibitor atomoxetine [7, 8].

Attention is not the only cognitive process modulated by NE in PFC circuits. It is also known that working memory is highly dependent upon noradrenergic transmission in the PFC. Specifically, delay-related firing, an electrophysiological correlate of working memory in prefrontal neurons, occurs in response to a behaviorally relevant stimulus and persists in its absence until reward can be retrieved. This type of activation of prefrontal neurons is potentiated by activation of the *α*_2A_ receptor and diminished by its antagonists, which improve and impair working memory, respectively [71]. Interestingly, NE has a high affinity for the *α*_2A_ receptor and is therefore engaged during low to moderate levels of NE and LC activation. When prefrontal NE concentration increases due to elevated LC discharge, as might occur during stress, the lower affinity *α*_1_ receptor becomes engaged, inhibiting prefrontal cortical function and working memory.

A potential mechanism for this is through *α*_1_ receptor-mediated long-term depression (LTD) in PFC synapses [72], which has been associated with improvement in measures of behavioral flexibility [73]. It has been proposed that this switch allows lower order sensorimotor cortical areas to guide behavior with little modulation by prefrontal operations [45, 50]. Inhibition of PFC and cognitive functions such as working memory and sustained attention might be beneficial to animals under certain circumstances, such as during stress for promoting behavioral flexibility. In this way, attentional reserves can be dissociated from specific stimuli and reallocated to others in the environment that facilitates escape from the stressor under guidance by more posterior cortical areas or to identify novel behavioral contingencies.

Disinhibition of PFC is known to impair behavioral flexibility [74], a major cognitive function which is disrupted in schizophrenic patients [75]. Research has shown that *α*_1_ receptor-dependent LTD is impaired in an animal model of schizophrenia [76], which could potentially account for some of the perseverative behaviors seen in this patient population. Therefore, it seems that “optimal” cognition is context-dependent and may be heavily modulated by NE. Furthermore, in some circumstances, enhancement of working memory and sustained attention might be beneficial and behavioral flexibility maladaptive, while in other circumstances, the opposite would be true. The switch between these two behavioral modes appears to be at least partially dependent on differential engagement of *α*_1_ and *α*_2A_ receptors. This notion is supported by evidence that suggests transmission at these receptors might be impaired in diseases characterized by cognitive deficits such as schizophrenia and ADHD [46].

Despite the important role that NE has in PFC function, particularly at the *α*_2A_ and *α*_1_ receptors, activation of the *β* receptor in PFC produces minimal effects on behavior and circuit operations [34]. Activation of the *β* receptor in the hippocampus, however, plays a major role in hippocampal-dependent cognitive function. Specifically, activation of the *β* receptor is necessary for both contextual and spatial memory consolidation and retrieval [15–17, 47, 48], as well as contextual fear memory [77]. Interestingly, however, it has been shown that mice genetically lacking NE display normal fear memory [78], suggesting that in its absence, other transmitter systems might play a compensatory role to restore it. Research suggests that the activation of the *β* receptor increases neuronal excitability in the dentate gyrus, CA1, and CA3 [79–81] and facilitates learning by promoting both long-term depression and long-term potentiation in hippocampal synapses [18–20]. Which type of plasticity occurs seems to depend upon the degree of activation of the *β* receptor [82].

Less evidence exists for a role for *α*-adrenergic receptors in hippocampal function. However, the *α*_1_ receptor may play an opposing role in hippocampal synaptic plasticity and memory formation and recall. Local application of the *α*_1_ receptor antagonist prazosin into the dentate gyrus has been shown to increase the rate of learning of active avoidance. Conversely, this behavior was acquired more slowly when the *α*_1_ agonist phenylephrine was administered [83]. This may be explained in part by the observation that *α*_1_ receptor activation increases action potential generation in inhibitory CA1 interneurons, leading to inhibition of pyramidal cells [84]. Moreover, prazosin has been shown to limit memory deficits in a mouse model of Alzheimer's disease [85], further supporting the notion that activation of the *α*_1_ adrenergic receptor (AR) is detrimental to hippocampal-dependent mnemonic processes. Interestingly, LC degenerates in Alzheimer's disease [27, 29, 44], which due to the well-established role of the *β* receptor in memory consolidation and recall as well as the particularly dense noradrenergic innervation of the hippocampus [18], likely contributes to some of the cognitive deficits displayed by this patient population. Importantly, manipulations that promote noradrenergic transmission are known to facilitate memory consolidation in normal aging patients as well as those showing mild cognitive impairment [86], suggesting that this transmitter system represents a viable target for the treatment of disease characterized by memory deficits. The strong evidence for the contribution of the LC/NE system to cognition in general through its actions at various receptor subtypes in PFC and hippocampus suggests that it represents a broad target for the treatment of the symptoms of various neurodegenerative, neuropsychiatric, and neurodevelopmental disease states, outlined below (Table 1).

## 3. Role of LC/NE System in Neuropathologies

### 3.1. Alzheimer's Disease

More than 35 million people worldwide live with dementia (5.5 million in the United States), and the number is set to almost double every 20 years. Moreover, the rate of undetected dementia is about 61.7% [87]. Alzheimer's disease (AD) is the most common form of dementia. Its pathogenesis includes amyloid plaque formation [88] and accumulation of tau protein [89] with subsequent oxidative and inflammatory brain damage [90, 91]. LC is a major contributor to AD progression: both preclinical studies of animal models of AD and clinical studies on postmortem human brain tissue [92] report decreased LC volume and numbers of tyrosine hydroxylase-positive LC cells. One proposed mechanism for forebrain NE loss in AD is a decrease in somatostatin receptor-2 (SSTR2) in LC neurons [93]. Significant somatostatin and SSTR-2 reduction has been described in normal aged brains across species and in human AD brains. Accordingly, a preclinical study of SSTR-2 knockout mice has revealed degeneration of noradrenergic axons with swollen varicosities and cluster-like structures [94], likely the result of accumulation of intra-axonal material due to impaired axonal transport [93].

A more prevalent hypothesis for LC degeneration and NE loss in AD is that accumulation of neurofibrillary tangles, comprised of abnormally phosphorylated tau protein, contributes to LC cell death and degeneration. Normal tau is a soluble protein which promotes assembly of tubulin, stabilizes microtubules, and facilitates axonal transport [89]. Hyperphosphorylated tau self-assembles into cytotoxic insoluble paired helical filament structures, contributing to cell death and impaired axonal transport [90]. According to Braak's classification of AD, LC plays a critically important role in pathogenesis of AD by undergoing accumulation of tau protein earlier than in other brain regions, which then serves as a primary source of the protein to the brain [95], causing neuronal degeneration and negatively impacting cognitive function. Animal models also demonstrate the accumulation and spreading of tau in LC. Stereotaxic injection of a bacterial vector carrying the human tau isoform into rodent LC leads to ipsilateral as well as contralateral accumulation of tau protein in the LC beginning the second week after injection, with frontal cortex becoming tau-positive in three months. Interestingly, maximum tau accumulation was observed from one to three months with decreases after six months due to loss of LC neurons [96]. This study did not find evidence of tau accumulation in hippocampal regions even six months after injection [96], despite the described accumulation of tau in human brains with AD [97]. This may suggest that LC cells innervating frontal cortex are distinct from those innervating hippocampus and uniquely susceptible to tau toxicity. This hypothesis is supported by prior observations from our laboratory showing an anatomically and functionally distinct projection from LC to frontal cortex [98].

Furthermore, evidence suggests that LC degeneration in AD affects mostly rostral cortically-projecting neurons and spares the caudal region [99], bolstering the argument for some degree of heterogeneity in LC susceptibility to AD pathogenesis. Identification of unique factors or markers that are expressed by LC neurons that are susceptible to AD pathogenesis may be informative of ways to limit or prevent LC degeneration experimentally, as well as for preventative/therapeutic purposes. If loss of LC cells that innervate frontal cortical regions contributes to the impaired cognition and dementia seen in AD, then limiting their damage might help to prevent the development of these symptoms. Notably, NE has been shown to be neuroprotective by reducing oxidative stress [100]. Thus, the early loss of NE that would follow LC degeneration might exacerbate later cognitive decline by failing to limit frontal cortical cell death.

Despite the lack of evidence for hippocampal tau in the aforementioned study, it is important to note that a body of data exists which suggests the necessity of proper hippocampal NE for normal cognition. Specifically, immunotoxic ablation of LC in young rats results in reduced proliferation of progenitor cells in hippocampal dentate gyrus [101]. Furthermore, systemic administration of the neurotoxin DSP-4, which selectively destroys noradrenergic neurons, dramatically depletes hippocampal NE [42, 102, 103]. Hippocampal tau accumulation and noradrenergic axonal degeneration in human AD patients could contribute to hippocampal dysfunction and cognitive decline, due to the role of NE in long-term potentiation and synaptic plasticity [18]. Impairment of hippocampal NE transmission due to accumulation of hyperphosphorylated tau and axonal degeneration could manifest as impairments in hippocampally dependent cognition and memory. In support of this hypothesis, it has been shown that genetic overexpression of amyloid precursor protein and presenilin-1, or genetic deletion of Ear2, which promotes LC development [104, 105], both modestly impair hippocampal long-term potentiation and spatial memory. These two genetic modifications together, however, act synergistically to further impair these functions [104]. Collectively, these data confirm a permissive role for LC-derived NE in hippocampal neurogenesis and function.

In addition to its role as a source of tau to the brain, LC cell death might further exacerbate AD progression by limiting forebrain concentrations of NE. Preclinical investigations of the role of LC in AD pathogenesis suggest a neuroprotective and anti-inflammatory role for NE [106]. In vitro, NE increases IkB*α* [107], an inhibitor of proinflammatory transcription factor NF-kB. Inflammation is an important component in AD pathogenesis and promotes microglial activation [108], complement cascade, and inflammatory cytokine release [109] with nitric oxide activation. Animal studies have shown anti-inflammatory effects of pretreatment with a *β*-adrenergic receptor agonist [109] and *α*_2_ receptor antagonist [110] on inflammation development in neurons after injection of *β* amyloid into LC-ablated animals. It is important to note that some discrepancies exist between preclinical animal studies and clinical human studies: LC in animal AD models tends to be involved in late stages of pathology, compared to its degeneration in early disease progression in humans. Therefore, the development of better AD animal models with primary effects on the noradrenergic system occurring early in pathogenesis will be an important factor in better understanding the sequence of pathologic processes that occur in the human AD brain.

### 3.2. Parkinson's Disease

The second most common cause of dementia are pathologies accompanied by Lewy body formation, including Parkinson's disease (PD) [111] and dementia with Lewy bodies. Considering the similar underlying pathophysiology, the effects of these diseases on LC will be considered together. The prevalence of PD is 200–300/100,000 [112], and even though motor symptoms are the primary concern, dementia occurs in as many as 80% of cases [113]. Despite weaker evidence for dysfunction of the noradrenergic system than the dopaminergic system in PD, it is critically important to consider the role of this transmitter system for a complete understanding and better management of cognitive impairment and emotional symptoms in PD patients that often accompany the more characteristic motor deficits seen in these diseases. The hallmarks of PD are degeneration of dopaminergic substantia nigra neurons and accumulation of *α*-synuclein in the form of Lewy bodies [114]. *α*-synuclein is a protein abundant in presynaptic terminals. It has been proposed that *α*-synuclein induces polymerization of purified tubulin into microtubules [115] and assists in vesicle fusion with presynaptic terminals and vesicle recycling [116]. Mutations in the *α*-synuclein gene could cause it to polymerize into filaments, which, with time, leads to nerve degeneration [117]. Indeed, postmortem studies of PD brains have described a loss noradrenergic neurons in LC and subcoeruleus in general, without topological preferences in contrast to LC degeneration in AD [99]. Furthermore, LC neuronal degeneration is accompanied by loss of overall structure, swollen cells with accumulated Lewy bodies, and short and thin dendrites [118, 119]. These neuronal changes collectively lead to overall decreased concentration of NE through the brain impacting LC terminal fields such as PFC and hippocampus thereby detrimentally affecting cognition.

According to Braak staging [120], LC accumulates *α*-synuclein and degenerates prior to substantia nigra, which exacerbates degeneration of the nigrostriatal pathway due to loss of neuroprotective and trophic influences of NE [100]. The neuroprotective action of NE is evidenced by the 1-methyl-4-phenyl-1,2,3,6-tetrahydropyridine (MPTP; a potent neurotoxin which destroys dopaminergic neurons) model of PD: compared to controls, mice with increased concentrations of NE in the central nervous system are less susceptible to neurotoxic effect of MPTP [121]. Another study in monkeys revealed differences between animals with and without LC lesions after MPTP. Animals with LC damage had persistent Parkinsonian motor signs by nine weeks, and postmortem histology demonstrated severe neuronal loss in substantia nigra (SN) as well as profoundly decreased dopamine content, while animal without LC destruction mostly recovered by six to nine weeks after early PD symptoms, and showed only moderate loss of SN neurons [122]. These observations further confirm the protective role of NE on the nigrostriatal pathway. What is more, the protective effect of NE seems to occur through *α*_2A_ receptors: when blocked by the specific antagonist yohimbine, MPTP toxicity in the SN is exacerbated [123].

There is also substantial evidence that LC degeneration contributes directly to the cognitive and emotional disturbances experienced by PD patients that precede dopaminergic motor deficits. Simple sensory discrimination is impaired in PD patients early in disease progression [56, 124], as well as various aspects of behavioral flexibility [125, 126] which have been shown in animal studies to be dependent upon intact noradrenergic signaling in PFC [7, 8, 53]. Evidence for a neurochemically complex etiology of cognitive impairment seen in PD comes from observations that treatment with dopamine agonists alone can ameliorate some, but not all behavioral deficits seen in this patient population. Behaviors which are not improved by dopaminergic agonists include attentional set shifting, task switching abstract rules, pattern and spatial recognition memory, associative learning, and verbal memory [127]. Given the importance of NE in modulating these cognitive functions [55], and extradimensional set shifting in particular, the LC/NE system represents a viable target for treatment options for sensory and cognitive deficits that accompany the more canonical motor deficits seen in PD. Mild cognitive decline or other behavioral markers for noradrenergic dysfunction that present prior to PD motor symptoms such as impaired sensory discrimination [56] might therefore be used as surrogate markers for early PD development, allowing for early intervention and preventative strategies to improve health outcomes within this disease population.

### 3.3. Attention-Deficit/Hyperactivity Disorder

Attention-deficit/hyperactivity disorder (ADHD) is a clinically heterogeneous and multifactorial disorder characterized by prevailing symptoms of poor attention, impaired working memory, and hyperactivity and/or impulsivity [44]. Worldwide prevalence is about 5.29% among children with higher levels in North America and Europe [128] and 3.4% among adults globally [129]. While ADHD presents with little evidence for neuroanatomical changes, and diagnostic criteria are limited to behavioral signs, there are animal models in which pathogenesis is explained by a lack of phosphoinositide 3-kinase PI3K*γ* [130]. This enzyme has been shown to play an important role in NMDA synaptic plasticity [131]. Knockout of PI3K*γ* in animals lead to increased levels of cAMP and subsequent stimulation of the transcription factor CREB, which regulates the ratio of NA to DA in PFC and striatum [132]. Impairment of the NE/DA ratio could lead to dysregulation of synaptic plasticity, which in turn promotes behavioral flexibility [133]. The pathogenesis of ADHD is connected to imbalances in dopaminergic and noradrenergic monoamine systems, which are therefore widely used as effective targets for its treatment [134].

Another proposed mechanism for ADHD is impaired NE transporter (NET) function. Drugs that inhibit NET such as methylphenidate and atomoxetine have been shown to improve sensory signal processing and behavioral outcomes in animals performing signal detection, flexible attention, and sustained attention tasks [8–10, 135]. However, it has been shown that availability and distribution of NET is not changed in ADHD patients according to a PET scan study [136], suggesting a potentially complex etiological origin for disease symptoms. Regardless, behavioral and pharmacological evidence still suggests that noradrenergic transmission is an effective therapeutic target for the treatment of ADHD symptoms and impaired working memory specifically at the presynaptic *α*_2A_ receptor. Administration of the *α*_2A_ agonists clonidine and guanfacine both improve behavioral and electrophysiological indices of working memory through inhibition of cAMP and strengthening of the functional connectivity of PFC networks [71, 135, 137], measures which are impaired by receptor antagonists such as yohimbine [71, 135, 138].

Dysfunctional noradrenergic transmission within the PFC seems to be implicated in the hyperactivity and attention impairment seen in ADHD. Motor hyperactivity can be induced in nonhuman primates through local administration of the *α*_2A_ antagonist yohimbine [138], providing further evidence for a specific role of prefrontal NE in modulating aberrant behavior in ADHD. Additional evidence for this hypothesis comes from observations that selective ablation of prefrontal noradrenergic fibers promotes perseveration and behavioral rigidity [7, 8], hallmarks of ADHD which are alleviated by inhibitors of NE reuptake [8]. Collectively, these observations lend support to the hypothesis that prefrontal NE is at least targetable for the treatment, if not directly related to the development of ADHD-like behavioral symptoms.

### 3.4. Schizophrenia

Schizophrenia (SCZ) is a debilitating mental illness which affects roughly 0.5% of the population and is considered to be one of the top ten causes of disability by the World Health Organization [139]. The illness is most effectively treated with antipsychotic drugs [140]; however, the variation of efficacy and side effects between patients for any one drug is substantial [141] and highlights our poor understanding of the disease. SCZ is characterized by positive symptoms such as delusion, hallucinations, and disordered thought and by negative symptoms such as blunted affect, inattention, and abulia [142]. While a major prevailing hypothesis is that altered dopaminergic and/or glutamatergic signaling contribute to SCZ development and etiology [143], there is evidence that the LC-NE system also plays a role in its major symptoms [144]. Specifically, on the basis of pharmacological, biochemical, and psychophysiological evidence, it has been proposed that both positive and negative symptoms may be the result of dysregulation of NE. NE has been found to be elevated in both the blood plasma [145] and cerebrospinal fluid of patients with SCZ, especially those with positive symptoms such as paranoia [145–147]. Postmortem studies have also reported increased markers for NE in the brains of schizophrenic patients [148–150]. Moreover, symptoms are often comorbid with insomnia [151], which is associated with the LC-NE system due to its role in promoting wakefulness [152].

In general, drugs that decrease, either directly or indirectly, noradrenergic transmission in the brain, such as *α*-methyldopa [153], clonidine [154], and propranolol, [155] tend to ameliorate positive symptoms. *α*-methyldopa, which interferes with the synthesis of both norepinephrine and dopamine, in conjunction with chlorpromazine, a D2 dopamine receptor antagonist, has been shown to be effective at treating schizophrenic behaviors as measured by the Inpatient Multidimensional Psychiatric Scale (IMPS) and the Brief Psychiatric Rating Scale (BPRS) [153]. Clonidine, in patients with predominantly positive symptoms according to the New Haven SCZ Index, has proven effective to improve symptoms with equal efficacy as a standard dopaminergic antagonist neuroleptic, while simultaneously alleviating tardive dyskinesia [154]. It has also been shown to increase executive function in patients [156] and restore NE lesion-induced cognitive impairment in the frontal cortex in nonhuman primates [157]. Furthermore, propranolol improves scores on a modified BPRS similarly to chlorpromazine [155]. Conversely, drugs that tend to increase NE concentration, such as methylphenidate [158], cocaine [159], yohimbine [160], and desipramine [161], tend to worsen positive symptoms. Specifically, methylphenidate, a catecholamine reuptake inhibitor, triggered or exacerbated psychotic symptoms in SCZ patients who were in the active phase of their disease [158]. Cocaine, another catecholamine reuptake inhibitor, induced paranoia as reported by cocaine-dependent patients [159], and yohimbine, an *α*_2A_ adrenergic antagonist, caused dysphoria after administration that was not seen in healthy subjects [160]. After 4 weeks of receiving desipramine, a norepinephrine reuptake inhibitor, patients performed more poorly on the BPRS hallucinatory behavior item [161].

Atypical antipsychotics have varying effects on the NE system. Clozapine and olanzapine, dopamine antagonists, have been shown to increase firing rates [162, 163] and Fos expression in the LC [164]. This observation, coupled with the clozapine's high affinity for the *β* receptor (*K*_i_ > 5000), indicates that it may promote strong actions on noradrenergic signaling in the brain. Additionally, the NET inhibitor reboxetine has been shown to selectively increase prefrontal levels of DA, which might contribute to some DA abnormalities in SCZ [163]. Further evidence for the role of interactions between dopaminergic and noradrenergic transmission in PFC comes from the observation that risperidone, an antagonist of dopamine and serotonin receptors, improves working memory function, an effect which is blocked by propranolol [164]. This suggests that risperidone may improve cognitive function by indirectly modulating noradrenergic signaling. Collectively, these findings show that while a constellation of transmitter actions contribute to cognitive function and dysfunction, modulation of the noradrenergic system in particular seems to promote myriad positive and negative therapeutic outcomes in the schizophrenic patient population.

Unlike the general consensus for degeneration of noradrenergic cell bodies and axons in AD and PD, anatomical evidence for an altered LC-NE system in SCZ is conflicting. Orbitofrontal cortices of a transgenic mouse model for SCZ that expresses a mutant version of a gene that predicts susceptibility in human SCZ patients (DISC1) were shown to contain shorter tyrosine hydroxylase (TH) positive fibers compared to wild-type mice [165, 166]. It is important to note, however, that these studies did not differentiate between NE and DA positive tyrosine hydroxylase profiles, so further investigation is necessary to determine the contribution of each of these transmitter systems to anatomical and behavioral changes seen in this animal model. However, DISC1 mutants display deficits in spatial working memory [167], which has also been linked to dysregulation of the NE system [168]. Human studies of schizophrenic brains have yielded mixed results about anatomical and morphological changes that occur in the noradrenergic system. One postmortem study reported that LC cells from schizophrenic brains are 50% larger than in control brains [169], while a similar study conducted in the same year concluded that there is no such change of the LC in SCZ [170]. Lastly a postmortem study showed that iodoclonidine, a derivative of clonidine, bound more weakly to *β* receptors in the hippocampus in the brains of SCZ patients [171], suggesting altered noradrenergic signaling in these patients, but not necessarily structural changes to the LC/NE system. At present, there is no consensus regarding the abnormalities of the LC in SCZ patients. Because of the diversity of symptoms and drug efficacies among patients, it is likely that LC-NE aberrations may differ between patients.

Despite lacking evidence for anatomical changes in LC of SCZ patients and animal models, there is behavioral evidence for dysfunction of the noradrenergic system. A surrogate marker widely used for SCZ-like symptoms clinically and preclinically is prepulse inhibition (PPI). This is a phenomenon in which a weak nonstartling acoustic stimulus is presented prior to a stronger more salient stimulus, and the presentation of the former inhibits the behavioral reaction to the latter. Disruption of this behavior such that the reaction to the second pulse is not diminished by the presence of the first is a phenotypic marker of SCZ in both the clinical patient population as well as in animal models for SCZ such as DISC1 mutant mice [172–174]. PPI in mice has been shown to be restored by atomoxetine [175], a selective norepinephrine reuptake inhibitor, suggesting that lowered NE levels may be a contributor to decreased PPI. Conversely, pharmacological stimulation of LC with a number of drugs causes a deficit in PPI, which is reversed by administration of clonidine [176]. Collectively, while there is less evidence for anatomical changes that occur in the LC in SCZ patients, there are clear clinical and preclinical data that suggest at least functional alterations in the LC/NE system take place in this disease.

## 4. Conclusions

The LC, with its broad axonal arborization, and NE as a major modulatory monoamine in the CNS orchestrate the full range of their effects on cognitive processes through interactions with *α*_1_, *α*_2_, and *β* receptors which have varying affinities for NE and topographical localization in the CNS and promote distinct cellular and network effects through the brain [16, 71, 72, 85, 177–179]. While classically viewed as a mediator of waking and modulator of sensory signal detection, strong evidence now exists arguing for an important role of this transmitter system in cognition in both health and disease. It has been proposed that the LC/NE system contributes to cognition through its role in promoting wakefulness and improving sensory signal detection that are necessary for navigating through and learning in a complex dynamic world [12, 13]. There is also clear evidence for a trophic and neuroprotective role for NE throughout the brain that limits neurodegenerative processes in cognitive and motor circuits and promotes hippocampal neurogenesis necessary for mnemonic processes [100, 101, 106, 120]. A greater appreciation for the precise way in which NE promotes cognition in the normal brain is crucial to developing a better understanding of how noradrenergic transmission goes awry in diverse neuropathologies such as AD, PD, ADHD, and SCZ (Table 1). This includes anatomical and neuroplastic changes within the LC and its efferent network as well as alterations in adrenergic receptor levels and distributions throughout the brain, particularly in PFC and hippocampus. Such new information will lead to new approaches to restore normal LC function and improve cognition and quality of life for millions of afflicted individuals through better treatment strategies for these patient populations.

## Figures and Tables

**Table 1:** Clinical and preclinical anatomical and functional changes in NE/LC system and related cognitive symptoms of Alzheimer's disease, Parkinson's disease, ADHD, and schizophrenia.

	Alzheimer's disease	Parkinson's disease	ADHD	Schizophrenia
Functional/anatomical changes in NE/LC system	Decreased LC volume and cell numbers with a rostrocaudal gradient [27, 44, 99] Tau protein assembles in LC into neurofibrillary tangles starting in early adulthood [95, 96] Decreased CNS levels of NE [92, 93, 102–105] Impaired hippocampal neurogenesis [101] Impaired synaptic plasticity [104, 105]	General destruction of LC without pattern topography [27, 44, 99] *α*-synuclein accumulation in LC [120] Loss of protective effect of *α*_2_AR on NE/DA system [122, 123]	Imbalances in DA/NE monoamine systems [132] PI3K*γ* deficiency [130, 131] Impairment of NE transmission in PFC [71, 135, 137, 138] Improvement in behavioral symptoms by modulators of NE transmission [8, 10, 134, 135] Dysregulation of signaling at the *α*_2A_ receptor [71, 135, 137]	Orbitofrontal cortex in DISC1+/+ mice contain shorter tyrosine hydroxylase (TH) positive fibers compared to wild-type mice [165, 166] Impaired *α*_1_ receptor-dependent LTD [76] Decreased binding of adrenergic probe to *β*_1_AR [166, 173]

NE-related behavioral changes	Early sensory deficits: impaired olfactory discrimination [56, 124] Behavioral perseveration modulated by innervation of medial PFC [5, 7, 8, 53, 125, 126] Memory decline [18, 85, 104, 105, 124, 127]		Deficits in working memory, sustained attention, hyperactivity, impulsivity, behavioral flexibility [44, 134]	Positive symptoms associated with high NE state [142, 153–155, 169, 170] Impaired spatial working memory [158–161, 170]
